# MRI and CT compatible asymmetric bilayer hydrogel electrodes for EEG-based brain activity monitoring

**DOI:** 10.1038/s41378-024-00805-2

**Published:** 2024-10-29

**Authors:** Guoqiang Ren, Mingxuan Zhang, Liping Zhuang, Lianhui Li, Shunying Zhao, Jinxiu Guo, Yinchao Zhao, Zhaoxiang Peng, Jiangfan Lian, Botao Liu, Jingyun Ma, Xiaodong Hu, Zhewei Zhang, Ting Zhang, Qifeng Lu, Mingming Hao

**Affiliations:** 1 The Affiliated Lihuili Hospital of Ningbo University, Ningbo, Zhejiang 315046 P. R. China; 2 School of CHIPS, XJTLU Entrepreneur College (Taicang), Xi’an Jiaotong-Liverpool University, Taicang, Suzhou, Jiangsu 215400 China; 3 i-lab, Key Laboratory of Multifunctional Nanomaterials and Smart Systems, Suzhou Institute of Nano-Tech and Nano-Bionics (SINANO), Chinese Academy of Sciences (CAS), 398 Ruoshui Road, Suzhou, Jiangsu 215123 P. R. China

**Keywords:** Materials science, Engineering

## Abstract

The exploration of multi-dimensional brain activity with high temporal and spatial resolution is of great significance in the diagnosis of neurological disease and the study of brain science. Although the integration of electroencephalogram (EEG) with magnetic resonance imaging (MRI) and computed tomography (CT) provides a potential solution to achieve a brain-functional image with high spatiotemporal resolution, the critical issues of interface stability and magnetic compatibility remain challenging. Therefore, in this research, we proposed a conductive hydrogel EEG electrode with an asymmetrical bilayer structure, which shows the potential to overcome the challenges. Benefiting from the bilayer structure with different moduli, the hydrogel electrode exhibits high biological and mechanical compatibility with the heterogeneous brain-electrode interface. As a result, the impedance can be reduced compared with conventional metal electrodes. In addition, the hydrogel-based ionic conductive electrodes, which are free from metal conductors, are compatible with MRI and CT. Therefore, they can obtain high spatiotemporal resolution multi-dimensional brain information in clinical settings. The research outcome provides a new approach for establishing a platform for early diagnosis of brain diseases and the study of brain science.

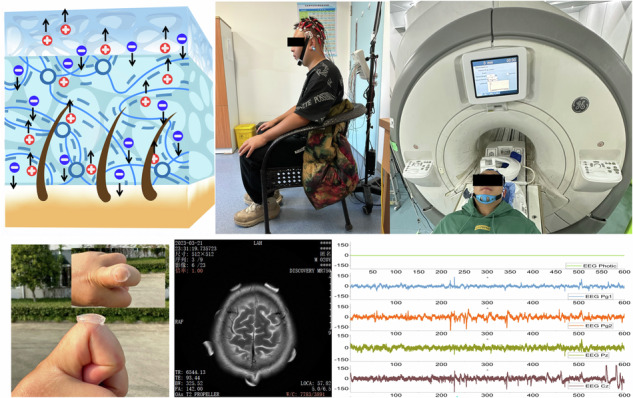

## Introduction

**Fig. 1:**
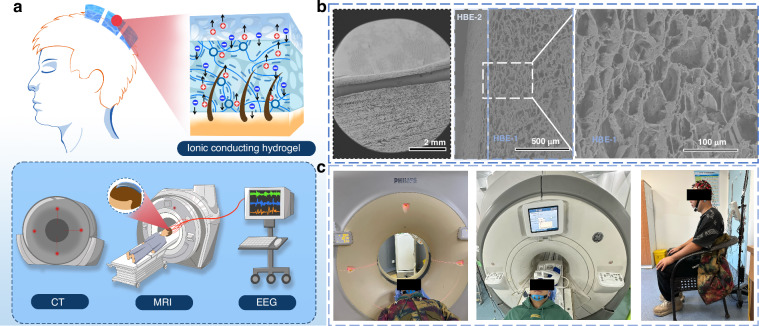
Schematic diagram of brain activity recording and microstructure morphology of the hydrogel EEG electrode. **a** Schematic diagram of brain activity recording. **b** SEM images of the hydrogel EEG electrode with the microstructure morphology of the two layers with different moduli. **c** Testing of the hydrogel EEG electrode in clinical applications, including CT and MRI environments

According to the World Health Organization (WHO), approximately one-third of the global population experiences neurological disorders, which are the leading causes of health loss and disability worldwide^1–3^. The diagnosis of neurological disorders is realized by the acquisition and analysis of electroencephalogram (EEG) signals due to their non-invasive property as shown in Fig. 1a. This approach is pivotal for managing neurological conditions and ensuring high-quality care, treatment, and rehabilitation services for patients^4–6^. However, EEG signals, characterized by frequencies from 0.5 to 100 Hz and amplitudes ranging from 1 to 200 μV, are prone to attenuation and distortion due to impedance mismatch when transmitted through subcutaneous tissues to the brain interface^7,8^. Moreover, human body movement generates additional interference to the EEG signals^9–11^. Consequently, the development of EEG electrodes capable of long-term signal collection is crucial for precision medical testing in neural diseases^12,13^.

Ionic conductive hydrogel, a soft material renowned for its excellent moisture retention and unique ion transport mechanism, is ideal for flexible devices and systems with high stretchability, conformal adhesion, noise filtration, and compatibility^14–22^. Consequently, several studies have been conducted on hydrogel electrodes^23–27^. Seung Hwan Ko’s team utilized PEDOT: PSS as a conductive filler to create high-stability conductive hydrogels that adhere to various substrates through laser-induced phase separation and interface structures. This method enables the selective transformation of conducting polymers into conductive hydrogels with wet conductivities of 101.4 S/cm and patterning with a spatial resolution as fine as 5 µm^28^. Yan and Zhang explored the development of a highly conductive, viscoelastic, and biocompatible hydrogel, capable of establishing an efficient and stable interface between human tissue and electrodes, yielding high-quality EEG signals via a non-invasive approach^29^. In collaboration with Suo, Wang introduced an implantable subdural cortical electrode with high transparency and low modulus, compatible with optogenetic equipment for multidimensional brain information detection in animal models^30^. However, the single-mode EEG signal suffers from low spatial resolution and has an impact on the feature identification accuracy. While CT and MRI offer higher spatial resolution for brain structural and functional signals, their strong magnetic fields significantly interfere with EEG sensors^31–35^. Consequently, The EEG electrodes, which is able to record EEG with CT and MRI images simultaneously have yet to be reported.

In this research, an asymmetrical bilayer hydrogel EEG electrode was proposed, featuring a lower modulus layer (HBE-1) and a higher modulus upper layer (HBE-2) in its structure as shown in Fig. 1b. The bilayer design endows the hydrogel with high conductivity, flexibility, and biocompatibility. Therefore, the high-quality EEG signals can be collected. In addition, benefiting from the metal-free property, the ionic conductive hydrogel is compatible with strong magnetic fields, which ensures its suitability in the operation of CT and MRI environments. As a demonstration, the integration of EEG with CT/MRI was achieved for the EEG signal and brain structural image recording as shown in Fig. 1c. This method facilitates high temporal and spatial resolution brain disease examinations. The research outcomes offer a novel approach for multi-dimensional brain information monitoring and the diagnosis of brain diseases.

## Result and discussions

### Mechanical properties of the hydrogel

**Fig. 2:**
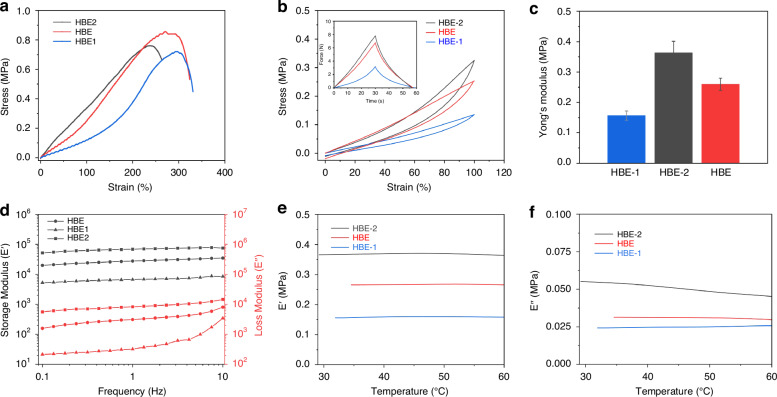
Mechanical properties of the hydrogel. **a** Stress-strain curves of HBE-1, HBE-2, and the bilayer HBE hydrogel at different tensile strains. **b** Cyclic stress-strain curves of HBE-1, HBE-2, and the HBE hydrogel at a tensile strain of 100%. **c** The Young’s Moduli of HBE-1, HBE-2, and the HBE hydrogel. **d** Rheological properties of HBE-1, HBE-2, and the HBE hydrogel at tensile strain frequencies of 0.1~10 Hz. **e**, **f** Rheological properties of HBE-1, HBE-2, and the HBE hydrogel at temperatures ranging from 25 to 60 °C

Figure 2 presents the mechanical performance of the hydrogel under different strain and temperature conditions. Figure 2a–c demonstrate that HBE-1 exhibits higher adhesion force within a lower strain range and has a low Young’s Modulus of 0.12~0.15 MPa. In contrast, HBE-2 shows lower flexibility and higher toughness within a larger strain range, with a high Young’s Modulus of 0.35~0.38 MPa. The bilayer structure of HBE has the advantages of both layers with Young’s Modulus between that of HBE-1 and HBE-2 (0.22~0.28 MPa). The storage modulus of HBE-1 in Fig. 2d indicates its high flexibility under dynamic conditions within the 0.1~10 Hz tensile vibration frequency. HBE-2 exhibits the highest storage modulus as well as a higher loss modulus, which indicates its higher rigidity and energy dissipation under dynamic conditions. The storage modulus and loss modulus of HBE are between those of HBE-1 and HBE-2. Figure 2e, f illustrate that HBE-2 shows the smallest variation in complex modulus under different temperatures. The results demonstrate its good stability under various temperature conditions. The static and dynamic mechanical performance of the hydrogel can provide evidence for the selection of appropriate hydrogel layers based on the requirements. The HBE has good resistance to water loss within 48 h (Fig. S1, Supporting Information). For applications with higher demands for comprehensive performance in clinical applications, the bilayer structure of HBE can be adopted to ensure the stable recording of signals. As a comparison, the property of PVA/PEG is shown in Fig. S2, Supporting Information.

### Adhesion properties of the hydrogel

**Fig. 3:**
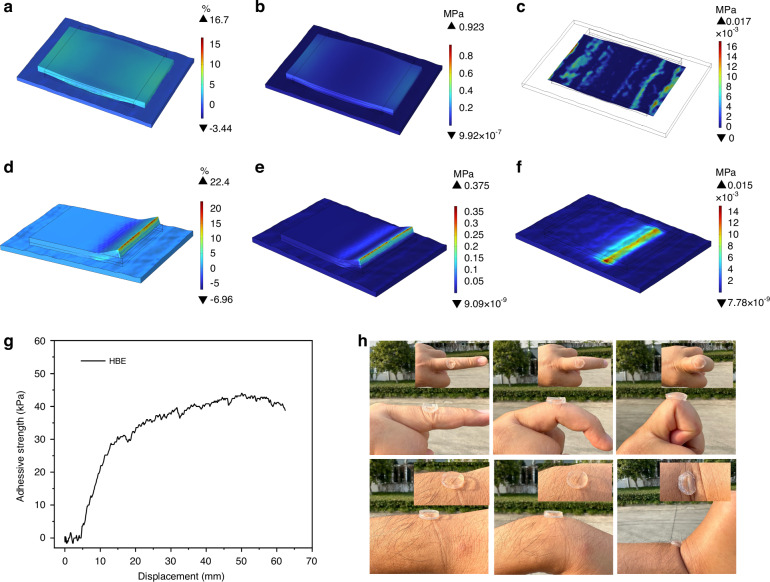
Adhesion performance of the hydrogel. **a**–**f** COMSOL simulation of the adhesion stress distribution of the hydrogel on the surface of the skin. **g** 90-degree peel adhesion strength test. **h** Adhesion optical images on the wrist and fingers of a human subject

The adhesion property of the hydrogel electrode is crucial for stable and reliable EEG signal monitoring, which is the fundamental requirement for accurate data collection and comfortable long-term wear. Therefore, the adhesion model of the hydrogel electrode on the surface of the skin was established by COMSOL. The adhesion process of the electrode on the skin surface and the distribution of adhesion strength were investigated and shown in Fig. 3a–f. In detail, Fig. 3a, d show the deformation of the hydrogel on the skin under tension, which demonstrates that the hydrogel undergoes minimum tensile deformation at the center of the skin strain according to simulation results. In detail, as shown in Fig. S3A, Supporting Information, the hydrogel is conformally contact with the pig skin in the absence of external forces. At this stage, due to the lack of external stress, no significant stress is observed. Fig. S3B, C show the deformation with small strain and the deformation at both ends of the hydrogel is larger than that in the middle. This phenomenon can be attributed to several factors. Firstly, external force is not instantaneously and uniformly distributed due to the flexibility of the skin and the viscoelastic properties of the hydrogel. In addition, the deformation depends not only on the magnitude of the applied stress but also the duration of external force. Under small strain, the hydrogel exhibits noticeable viscoelastic behavior, which means that the deformation process is slow. As a result, the stress initially concentrates at the ends, which causes significant deformation at the ends. However, the middle of the hydrogel shows minimal or almost no deformation due to delayed stress transmission and uneven distribution. This result is consistent with the stress distribution in COMSOL simulation.

Figure 3b, e illustrate the stress distribution and adhesion strength changes resulting from the stretch of the hydrogel. The results indicate that no significant impact was observed on the contact area with the skin surface. Figure 3c,f demonstrate the effect of the tensile deformation of the hydrogel. Simulation results show that the hydrogel exhibits uniform adhesion stress distribution on the skin surface which enables the stable attachment to biological tissue without significant stress during stretching.

A 90-degree peel test was conducted using the Instron universal material testing machine at a loading rate of 5 mm/min. The peel adhesion strength of the hydrogel gradually increases within the displacement range of 0 ~ 60 mm and reaches a maximum value of ~45 kPa as shown in Fig. 3g. The peel test results indicate that the hydrogel exhibits high peel adhesion strength and maintains stable adhesion performance within a large displacement range. In addition, the hydrogel adhered to the wrist and fingers of the subjects to investigate the adhesion property during the bending and stretching states as shown in Fig. 3h. The findings demonstrate that the hydrogel can stably adhere to the wrists and fingers of individuals during movement, indicating its potential for long-term wearable and flexible systems.

### Electrical properties of hydrogel-based EEG electrodes

**Fig. 4:**
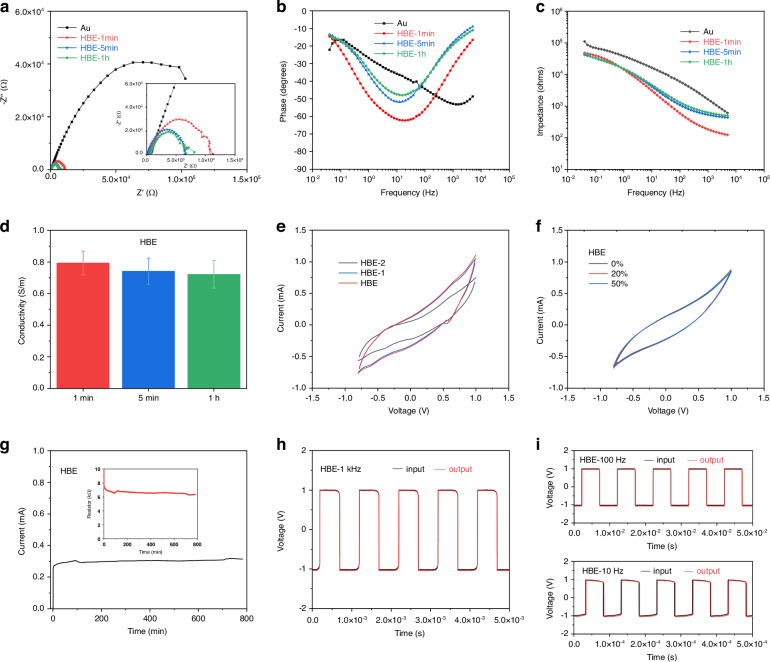
Electrical properties of the hydrogel-based EEG electrodes. **a**–**c** EIS spectra, phase angle, and frequency response of HBE, HBE-1, and HBE-2. HBE-1 is the hydrogel with low modulus and HBE-2 is the hydrogel with high modulus. HBE is the bilayer hydrogel electrode. **d** The conductivity of the HBE electrode at different times. **e** Cyclic voltammetry curves of HBE, HBE-1, and HBE-2. **f** Cyclic voltammetry curves of HBE under different strains. **g** long-term stability of the hydrogel. **h**, **i** Input and output voltage waveforms of HBE with different frequencies

The electrical impedance of the hydrogel-based EEG electrodes is also a crucial parameter in signal fidelity and reliability, which is important for neurophysiological studies. Figure 4a shows the electrical impedance spectra (EIS) of Au, HBE, HBE-1, and HBE-2 electrodes. The impedance of the Au electrode is significantly higher than that of the HBE electrode, indicating the better ion conductivity of the HBE electrode. Figure 4b illustrates the phase angle variation with frequency. The phase angle of the HBE electrode approaches 90 degrees in the low-frequency range, suggesting stronger capacitive behavior. Figure 4c displays the impedance variation with frequency. The impedance of the HBE electrode is significantly lower than that of the Au electrode, reaching the lowest impedance value after 1 h. In Fig. 4d, the conductivity remains steady at ~0.7–0.9 S/m with no significant decrease in the conductivity observed within the following 1 h. Figure 4e presents the cyclic voltammetry curves of the HBE, HBE-1, and HBE-2 electrodes. The current response of the HBE electrode is significantly higher than that of the single-layer hydrogel electrode, HBE-2, indicating its superior comprehensive conductive performance. The cyclic voltammetry curves of the HBE electrode at different tensile strains of 0%, 20%, and 50% were performed to test the reliability of the electrode during movement. As depicted in Fig. 4f, no significant change was observed. Figure 4g demonstrates the long-term stability of the HBE electrode, showing that the electrode can work stably for more than 12 h. Figure 4h, i show the input and output voltage waveforms at different frequencies, indicating that the HBE electrodes can effectively respond to the input signals up to 10 kHz.

### Biocompatibility of the EEG electrodes

**Fig. 5:**
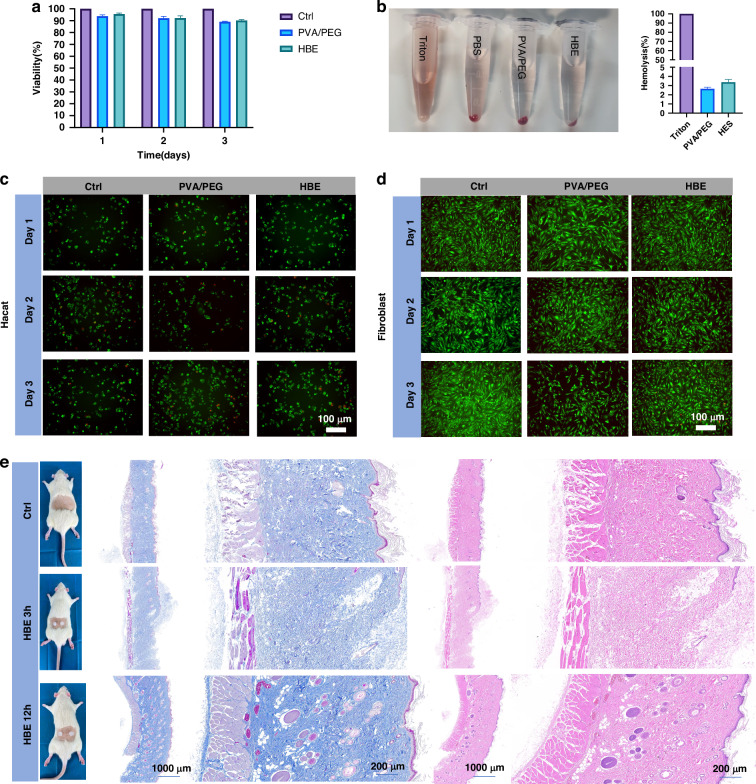
Cytocompatibility and hemocompatibility of the hydrogels. **a** Cell viability of L929 cells treated with different hydrogels for 1, 2, and 3 days. **b** Hemolysis photographs and Hemolysis ratio (%) of the hydrogels. **c**, **d** Live/Dead staining of Hacat and human dermal fibroblasts after being treated with different hydrogels for 1, 2, and 3 days. **e** Masson’s trichrome and H&E staining of skin tissues after being treated with HBE hydrogel for 3 and 12 h

Biocompatibility is a crucial factor for hydrogel electrodes to be used in biological systems without adverse effects. Therefore, cytocompatibility and hemocompatibility were investigated in our research. The cytocompatibility of the CCK8 cell viability assay is shown in Fig. 5a. Both the polyvinyl alcohol (PVA)/ Polyethylene glycol (PEG) and HBE materials sustained cell viability exceeding 90% over 1, 2, and 3 days, which is close to the PBS control group. This result indicates the minimal cytotoxicity towards L929 fibroblasts. In Fig. 5b, the hemolysis rate of the HBE material is notably lower than that of Triton X-100 (positive control), which indicates that no significant difference between the hydrogel and the PBS control group. Figure 5c, d present the results of the Live/Dead immunofluorescence staining. The HaCaT cells and human dermal fibroblasts within the PVA/PEG and HBE material groups exhibit vigorous growth, elevated cell density, and uncompromised morphology over 1, 2, and 3 days, without any appreciable discrepancies from the control group. This result confirms the exceptional cytocompatibility of the material. The hematoxylin and eosin (H&E) and Masson staining shown in Fig. 5e reveal that the skin tissue remained unperturbed after the application of the HBE to the rat dorsum for 3 and 12 h. No discernible influx of inflammatory cells or tissue perturbation was observed, indicating that the HBE material does not provoke skin irritation. To investigate the biocompatibility of HBE electrodes over extended periods, HBE hydrogel was implanted in the dorsal region of rats for a duration of 3 days. Histological analysis using H&E and Masson staining at 3 days post-implantation revealed no significant irritation to the subcutaneous tissue, and no adverse reactions were observed when compared to the control group (Fig. S4, Supporting Information). Furthermore, histological analysis using H&E staining at 3 days post-implantation demonstrated that there was no damage to the heart, liver, spleen, lung, or kidney, with normal structures preserved. This finding further supports the excellent biocompatibility of the HBE hydrogel electrodes (Fig. S5, Supporting Information).

### EEG signals recorded by hydrogel-based electrodes

**Fig. 6:**
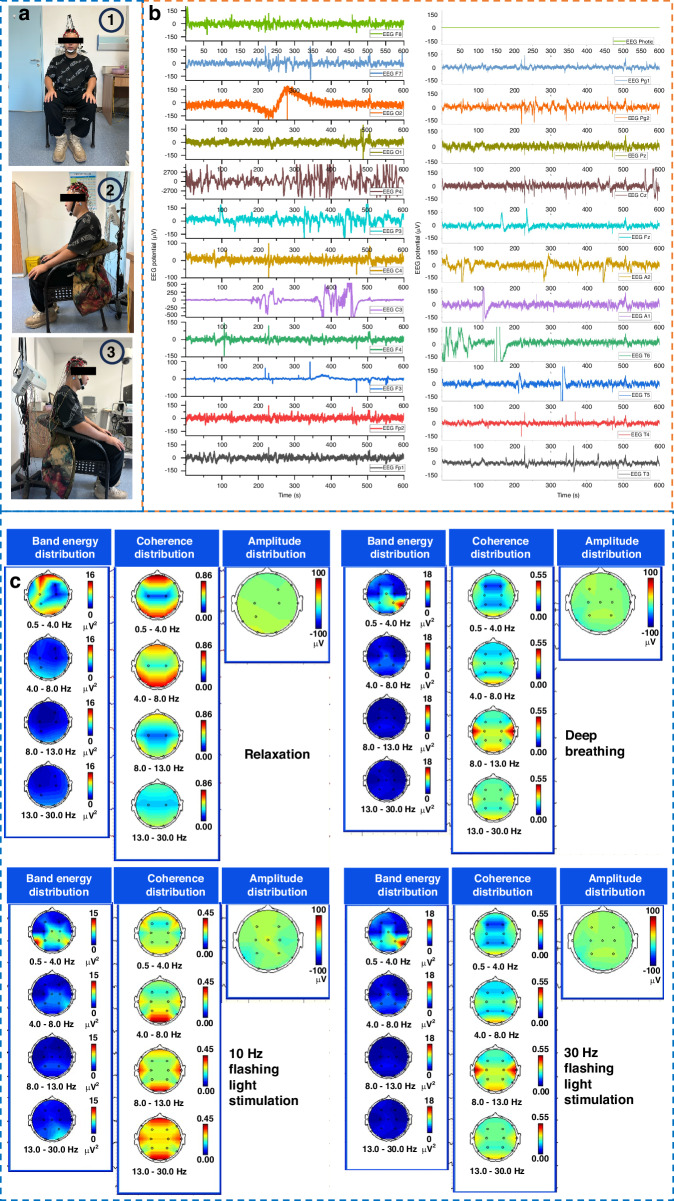
The testing of EEG signals from the subjects with the hydrogel EEG electrodes. **a**The test in a relaxed state, deep breathing, and flashing light stimulation. **b** EEG waveform depicting the brain electrical signals in different states. **c** Brain topography maps of the spatial distribution of brain electrical activity at different states (relaxation, deep breathing, 10 Hz frequency flashing light stimulation, and 30 Hz frequency flashing light stimulation)

After investigating the basic electrical and mechanical properties of the hydrogel, we conducted the EEG signal monitoring with the hydrogel-based electrodes and analyzed the EEG signals and brain topography. Figure 6a depicts the EEG signal recording in a relaxed sitting state, deep breathing in a sitting position, and exposure to a flashing light. From the energy spectrum, correlation distribution, and amplitude from EEG signals and brain topography in Fig. 6b, c, the EEG signals appear relatively stable, with minimal potential fluctuations across all channels, which indicates low brain activity of the subjects in the relaxed state. Representative EEG signals in 295 to 299 s with HBE electrodes are provided in Fig. S6, Supporting Information. The signal-to-noise ratio is calculated to be between 40–60 dB from the signals of channel F7 and T3. The analysis of brain topography and energy spectrum confirms that the signals concentrated in the 0.5–4.0 Hz (Delta waves) and 4.0–8.0 Hz (Theta waves) frequency ranges are mainly associated with relaxation. During deep breathing, noticeable rhythmic changes in the EEG waveforms are observed, which is consistent with the respiratory frequency. The energy spectrum in the 4.0–8.0 Hz (Theta waves) and 8.0–13.0 Hz (Alpha waves) frequency bands reflect the significant impact of deep breathing on brain electrical activity. In addition, there is a slight coherence increase than that in the relaxed state, which can be attributed to the enhanced brain activity. Under flashing light with a frequency of 10 Hz, a strong synchronized response is observed in the EEG waveforms. Significant enhancement in brain electrical activity appears in the 8.0–13.0 Hz (Alpha waves) and 13.0–30.0 Hz (Beta waves), accompanied by a noteworthy increase in coherence in the Alpha and Beta bands. The amplitudes are significantly enhanced due to the substantial brain electrical response to flashing light frequency. When the flashing frequency increases to 30 Hz, the EEG waveforms exhibit pronounced synchronized patterns with significant enhancement in brain activity observed in the 13.0–30.0 Hz (Beta waves) frequency band. The amplitudes also significantly increase, indicating a notable impact of the flashing frequency of the light on high-frequency brain electrical activity.

The analysis of EEG signals with the brain topography shows that the Delta and Theta waves are concentrated in the forehead, while Alpha and Beta waves are lower and evenly distributed across the scalp, signifying uniform electrical activity in these bands across the entire head. High coherence in the 0.5–4.0 Hz frequency range, especially in the forehead, indicates strong synchronized activity in the low-frequency range in the forehead. Low coherence in the 4.0–30.0 Hz frequency range suggests poor synchronization of electrical activity across different regions. The high amplitudes distributed in the middle and anterior regions of the head indicate greater electrical activity strength in these areas. The energy spectrum, coherence distribution, and amplitude of brain electrical activity under different states and stimuli in the research show potential for providing a comprehensive foundation for clinical examinations, diagnosis, and treatment recommendations for brain disease.

### Compatibility of hydrogel-based EEG electrodes in CT and MRI environment

**Fig. 7:**
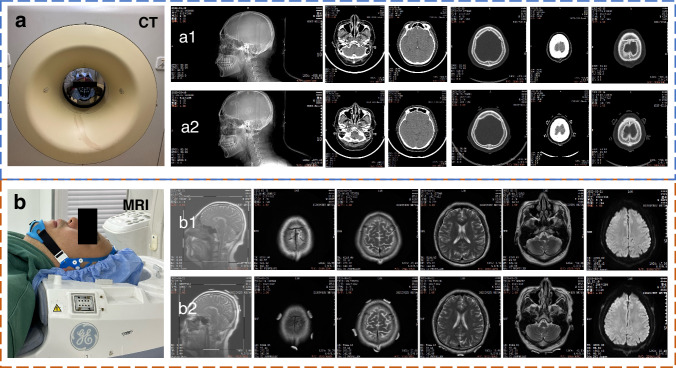
The CT and MRI of the brain. **a** a1 and a2 show the brain images of the subject undergoing CT scans with and without wearing hydrogel brain electrodes. **b** a1 and b2 present the brain images of the subject undergoing MRI scans with and without wearing hydrogel electrodes

Finally, the compatibility of the EEG hydrogel electrode with MRI and CT was conducted to demonstrate its potential application in clinical diagnosis. The CT images Fig. 7a1, a2 display the skull and brain structures. In contrast to the metal electrodes, no interference with the imaging is observed in the presence of the hydrogel electrodes. Fig. 7b1, b2 present the brain tissue structures with hydrogel electrodes, and almost no impact on the quality of the MRI images, such as magnetic resonance artifacts, was observed. The result indicates that the hydrogel electrodes can be used in both CT and MRI environments without the issues of artifacts as in traditional metal electrodes.

## Conclusion

The hydrogel, a soft and biocompatible material, has great potential in flexible and wearable systems for long-term monitoring of biological signals with comfort and no skin irritation. In this study, an asymmetric bilayer hydrogel was designed and synthesized for EEG electrodes. The top layer, characterized by a high modulus, ensures high conductivity, whereas the bottom layer, with a low modulus, improves wearing comfort and skin conformality. This bilayer design not only enhances interface contact but also maintains structural stability. High-quality EEG signals were successfully recorded in various states, including relaxation, deep breathing, and flash stimulation. In addition, the metal-free nature of the hydrogel electrodes allows compatibility with CT and MRI without generating artifacts. Consequently, the proposed hydrogel-based electrodes provide substantial benefits for monitoring complex brain activities and show a potential for simultaneous recording of multi-dimensional brain activity. The research findings lay a foundation for the comprehensive study of brain diseases and brain science.

## Materials and Methods

### Materials

Polyvinyl alcohol (PVA-117, Mw~145, 000), Polyethylene glycol (Average Mn 4, 000), and NaCl (Medical grade) were sourced from Shanghai Aladdin Industrial Corporation Co. Ltd. All other materials, unless specified, were commercially available and used as received. The deionized water utilized in the experiments consistently met Milli-Q grade standards with a resistivity of 18.2 MΩ.

### Synthesis of HBE and PVA/PEG Hydrogel

20 g PVA was dissolved in 80 mL of a 5% NaCl solution and the solution was stirred for 6 h at a temperature of 90 °C, labeled as Solution A. Similarly, 10 g PEG was dissolved in 80 mL of a 5% NaCl solution with a stirring of 6 h at 90 °C, labeled as Solution B. 50 mL of Solution A and Solution B were mixed and stirred for 6 h at 90 °C to obtain the HBE mixture. The HBE solution underwent degassing through ultrasonication at 60 °C for 1 h. Then, the mixed solution was carefully poured into molds and refrigerated at −20 °C for 12 h. The synthesized hydrogel is labeled as HBE-1. Subsequently, Solution A was cast on the surface of the frozen sample. After being stored at −20 °C for 6 h, the sample was naturally thawed for 12 h. The synthesized top layer hydrogel was labeled as HBE-2. The freeze-thaw cycle was repeated three times to form the bilayer structure hydrogel labeled as HBE. The preparation procedure for the PVA/PEG hydrogels was the same as that of HBE. However, the deionized water was used as the solvent instead of the 5% NaCl solution.

### Physical characterization of the hydrogel

The microstructures of the hydrogel of the dehydrated HBE were characterized using a scanning electron microscope (SU8010, Hitachi, Japan). To test the mechanical properties, the HBE, HBE-1, and HBE-2 were sliced as film specimens, with 50 mm in length, 20 mm in width, and 2 mm in thickness. Subsequently, the mechanical properties of these hydrogels were evaluated utilizing an Instron universal material testing apparatus (INSTRON 3365, USA). The initial separation between clamps was meticulously set at 20 mm, while the loading rate was regulated at 10 mm/min. To examine the skin adhesion characteristics of hydrogels, fresh pigskin was sliced into rectangular strips 200 mm in length, 40 mm in width, and 2 mm in thickness. These strips were then immersed in PBS to eliminate any residual contaminants. Similarly, the HBE was sliced into rectangular specimens with 100 mm in length, 20 mm in width, and 2 mm in thickness. The bonding area was configured at 70 × 20 mm^2^ with an initial separation distance of 10 mm. The tissue adhesion force of the hydrogel was measured using an Instron universal material testing apparatus with a loading rate of 5 mm/min. In the COMSOL Multiphysics simulation, the skin and hydrogel interface was simplified to a plane with surface roughness. The skin was set as the fixed bound while the interface between the hydrogel and skin was designated as a free surface in the model. Furthermore, the storage modulus (G′) and loss modulus (G″) of the HBE were assessed using a HAAKE MARS60 rheometer (Thermo-Fisher, USA). The oscillation frequency was from 0.1 Hz to 10 Hz, with a strain amplitude of 2%. In addition, the NETZSCH DMA 242 E dynamic thermomechanical analyzer was used to explore the dynamic behavior of G′ and G″ during a controlled heating process, spanning from 20 °C to 60 °C. The heating rate was calibrated at 5 °C/min with an oscillation frequency of 5 Hz and a uniform strain amplitude of 2%.

### Electrochemical characterization

Electrochemical impedance spectroscopy and cyclic voltammetry measurements were performed using a Gamry Reference 600 potentiostat/galvanostat/ZRA (Gamry Instruments, USA). EIS evaluations were performed within the frequency spectrum of 0.1–10 kHz. Cyclic voltammetry measurements were executed in the standard three-electrode configuration, utilizing Ag/AgCl (2 M KCl) as the reference electrode and an Au electrode, with a surface area of 1 cm², serving as the counter electrode. The signal transmission property was evaluated utilizing a TBS2000B digital oscilloscope (Tektronix, USA). Furthermore, the electrical properties, including the Current-Time curves of the HBE, were meticulously quantified with a digital Source Meter (Keithley 2400). All test samples were precisely dimensioned at 10 mm × 10 mm × 2 mm.

### Cell cultivation and biocompatibility

L929 fibroblast cells (L929, Noblebio, Hangzhou, China), human epidermal keratinocyte cells (HaCat, Noblebio, Hangzhou, China), and human dermal fibroblasts (HDFs, Procell, Wuhan, China) were cultivated in a humidified chamber maintained at 37 °C with an atmosphere with 5% CO_2_, utilizing DMEM (Procell, Wuhan, China) with 10% serum (Noblebio, Hangzhou, China). The cytotoxicity was quantified using the Cell Counting Kit-8 (CCK-8) assay (C0038, Beyotime Biotechnology, Shanghai, China). L929 cells were seeded at a density of 5 × 10^3^ cells per well in 96-well plates, exposed to the hydrogel-extracted solution. The solution was replaced with CCK-8 reagent after 1, 2, and 3 days, respectively. After a 2 h incubation at 37 °C, the optical density (OD) was measured at 450 nm. The cell viability was determined by the formulation

$${\rm{Cell}}\; {\rm{viability}}=\,({\rm{OD}}_{\rm{n}}-{\rm{OD}}_{\rm{c}})/({\rm{OD}}_{\rm{s}}-{\rm{OD}}_{\rm{c}})\times 100 \%$$

where OD_n_ represents the absorbance of the sample (hydrogel) group, OD_c_ is the absorbance of the blank group, and OD_s_ denotes the absorbance of the control group.

The cytocompatibility of various hydrogel formulations was evaluated using the Live/Dead fluorescence staining kit (R37601, Invitrogen, USA). The hydrogel extract was prepared by immersing 0.1 g of hydrogel in 10 mL of culture medium for 24 h. HaCat and HDFs were cultivated at a density of 2 × 10^4^ cells per well in 24-well plates, exposed to the hydrogel-extracted solution. Subsequently, it is stained with the dual-parameter dye with an incubation of 1 and 3 days, respectively. After a 30 min incubation, the cells were rinsed with phosphate-buffered saline (PBS) and examined under an inverted fluorescence microscope to discern live and dead cells.

The blood compatibility of the hydrogels was assessed through an in vitro hemolysis assay. Freshly mouse blood was centrifuged at 3000 RPM for 15 min at 4 °C and the pellet was washed five times with PBS. The blood cells were then diluted to a concentration of 5% w/v and cultivated with the hydrogels for 1 h at 37 °C. After centrifugation at 3000 RPM for 10 min, the supernatant was collected. The absorbance was measured at 540 nm. The hemolysis rate was calculated by the formula,

$${\rm{Hemolysis}}\; {\rm{rate}}=({\rm{OD}}_{\rm{n}}-{\rm{OD}}_{\rm{c}})/({\rm{OD}}_{\rm{s}}-{\rm{OD}}_{\rm{c}})\times 100 \%$$

where OD_n_, OD_c_, and OD_s_ are the absorbance of the supernatants with the treatment with the hydrogel, PBS solution, and deionized water, respectively.

To evaluate the in vivo histocompatibility of the hydrogel, the rats were used as control and HBE samples. The HBE material was attached to the dorsum of the experimental group rats, and the skin reactions were monitored for 3 and 12 h. After the removal of the material, a comprehensive examination was conducted utilizing hematoxylin and eosin (H&E), and Masson staining techniques to investigate the skin tissue and discern the inflammatory responses. To evaluate the long-term biocompatibility of HBE electrodes, HBE hydrogel was implanted in the dorsal region of rats for 3 days. Subsequently, skin tissue and major organs were harvested and analyzed using H&E and/or Masson staining.

### EEG, MRI, and CT

EEG signals were collected by a 32-channel system with the international 10–20 lead method and CPz was designated as the reference. The EEG signals were recorded using the eegoTM mylab (ANT Neuro, Enschede, Netherlands) at a sampling frequency of 1500 Hz. Both the reference and working electrodes employed in testing were the HBE hydrogel specifically developed in this study. The contact impedance between the brain electrode and scalp was observed in real time during EEG signal detection (Fig. S7, Supporting Information). MRI images and CT images of the subject, both with and without the HBE, were recorded using a clinical 3.0T-SIGNA™ Pioneer-70cm scanner (GE Healthcare, USA), and Brilliance CT Big Bore CT scanner (Philips, Netherlands).

## Supplementary information

Supporting Information
