# Supplementary material for: MRI and CT compatible asymmetric bilayer hydrogel electrodes for EEG-based brain activity monitoring
Source: Microsyst Nanoeng. 2024 Oct 29;10:156. doi: 10.1038/s41378-024-00805-2 (PMC11519644; doi:10.1038/s41378-024-00805-2)
Supplement: Supplementary file 1 — Supporting Information [file 41378_2024_805_MOESM1_ESM.docx]

MRI and CT Compatible Asymmetric Bilayer Hydrogel Electrodes for EEG-Based Brain Activity Monitoring

Guoqiang Ren^1^, Mingxuan Zhang^2^, Liping Zhuang^1^, Lianhui Li^3^, Shunying Zhao^1^, Jinxiu Guo^1^, Yinchao Zhao^2^, Zhaoxiang Peng^1^, Jiangfan Lian^1^, Botao Liu^1^, Jingyun Ma^1^, Xiaodong Hu^1^, Zhewei Zhang^1^, Ting Zhang^3*^, Qifeng Lu^2*^, and Mingming Hao^1*^

^1^ The Affiliated Lihuili Hospital of Ningbo University, Ningbo, Zhejiang 315046, P. R. China.

^2^ School of CHIPS, XJTLU Entrepreneur College (Taicang), Xi’an Jiaotong-Liverpool University, Taicang, Suzhou, Jiangsu 215400, China.

^3^ *i*-lab, Key Laboratory of Multifunctional Nanomaterials and Smart Systems, Suzhou Institute of Nano-Tech and Nano-Bionics (SINANO), Chinese Academy of Sciences (CAS), 398 Ruoshui Road, Suzhou, Jiangsu 215123, P. R. China.

Fig. S1. shows the water loss of the HBE within 48 hours.


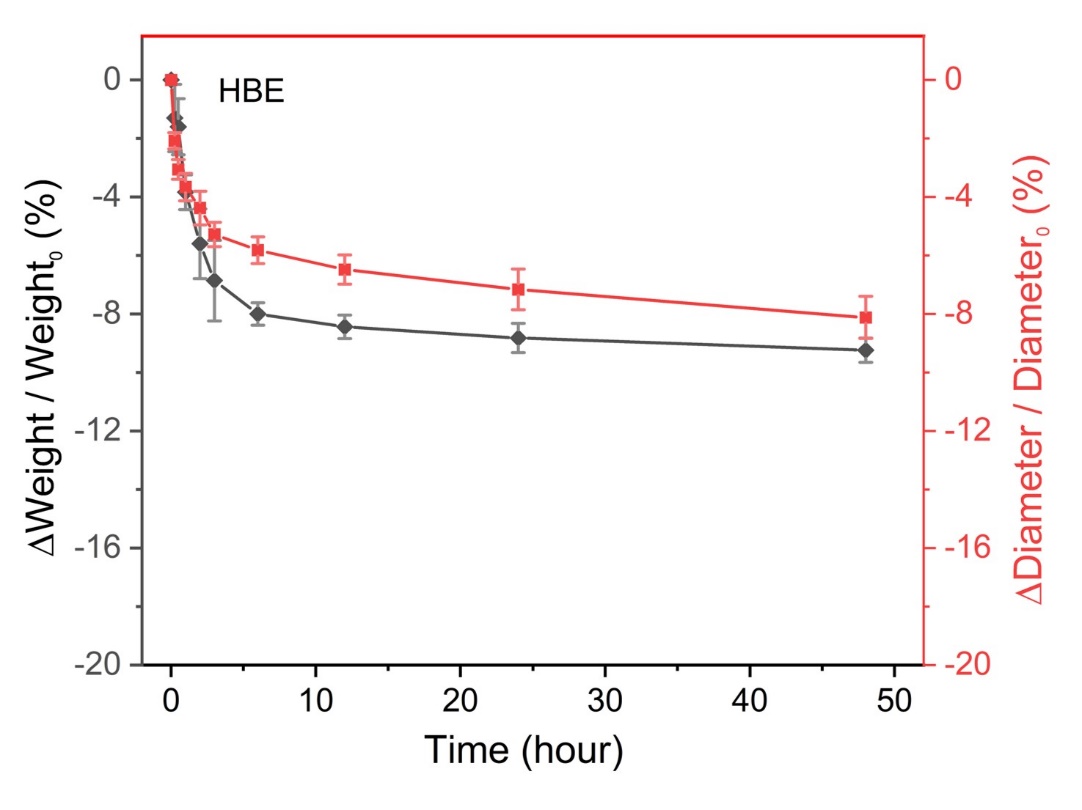


Fig. S1 Weight and diameter change of HBE over a 48-hour period

Fig. S2 shows that the PVA/PEG hydrogel prepared with deionized water have a higher mechanical strength and adhesive force than that with 5% sodium chloride solution. Also, a higher resistance is observed for PVA/PEG hydrogel due to lack of mobile ions.


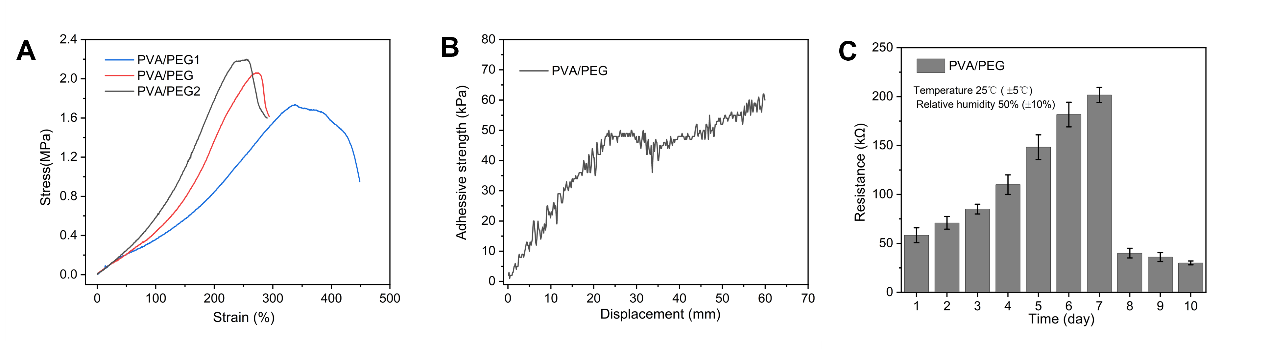


Fig. S2 The Stress-strain curves, adhesive strength and resistance of PVA/PEG.

Fig. S3 shows the deformation of HBE under small external force.


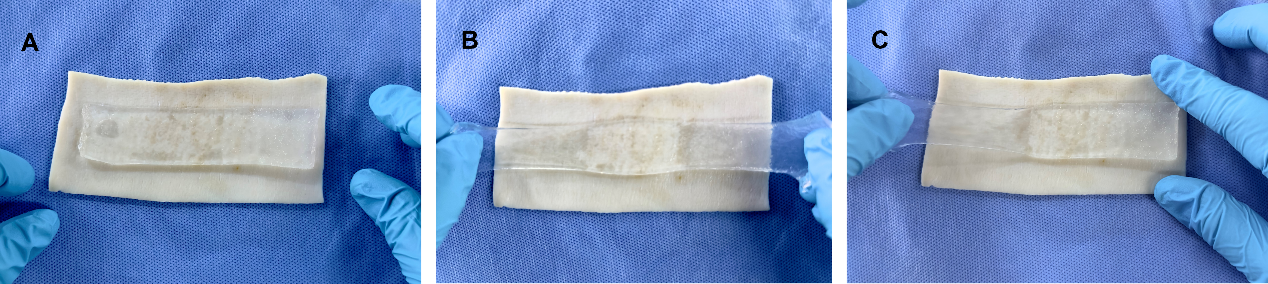


Fig. S3 The deformation of HBE under strain


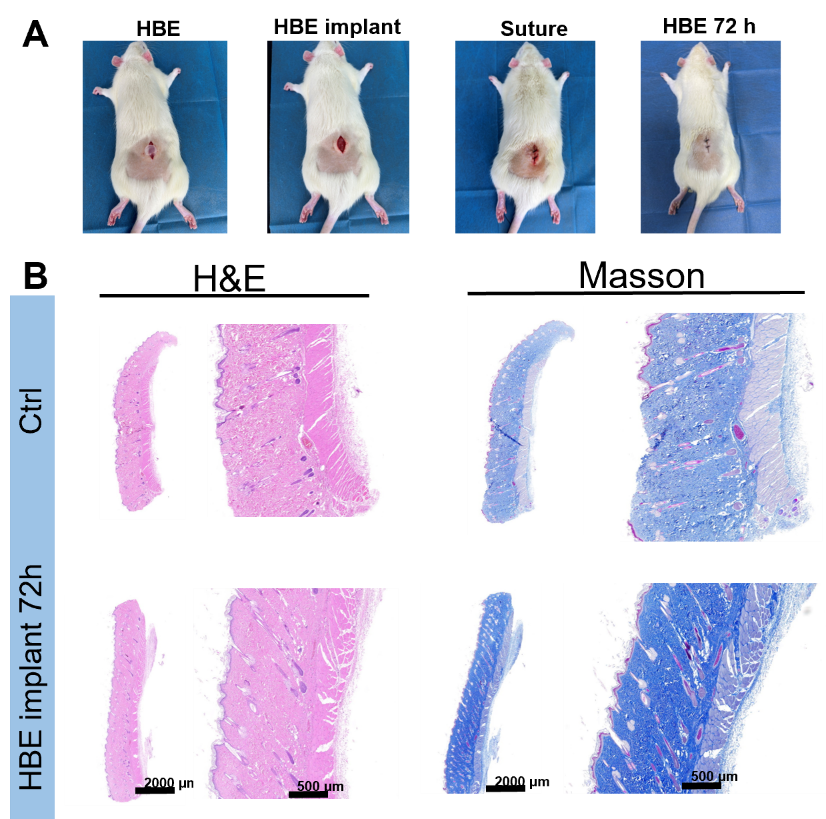
Fig. S4 shows the histological analysis using H&E and Masson staining at 3 days post-implantation revealed no significant irritation to the subcutaneous tissue, and no adverse reactions were observed when compared to the control group.

Fig. S4 The biocompatibility, allergenicity, and irritability result for the subcutaneous implantation of hydrogel.


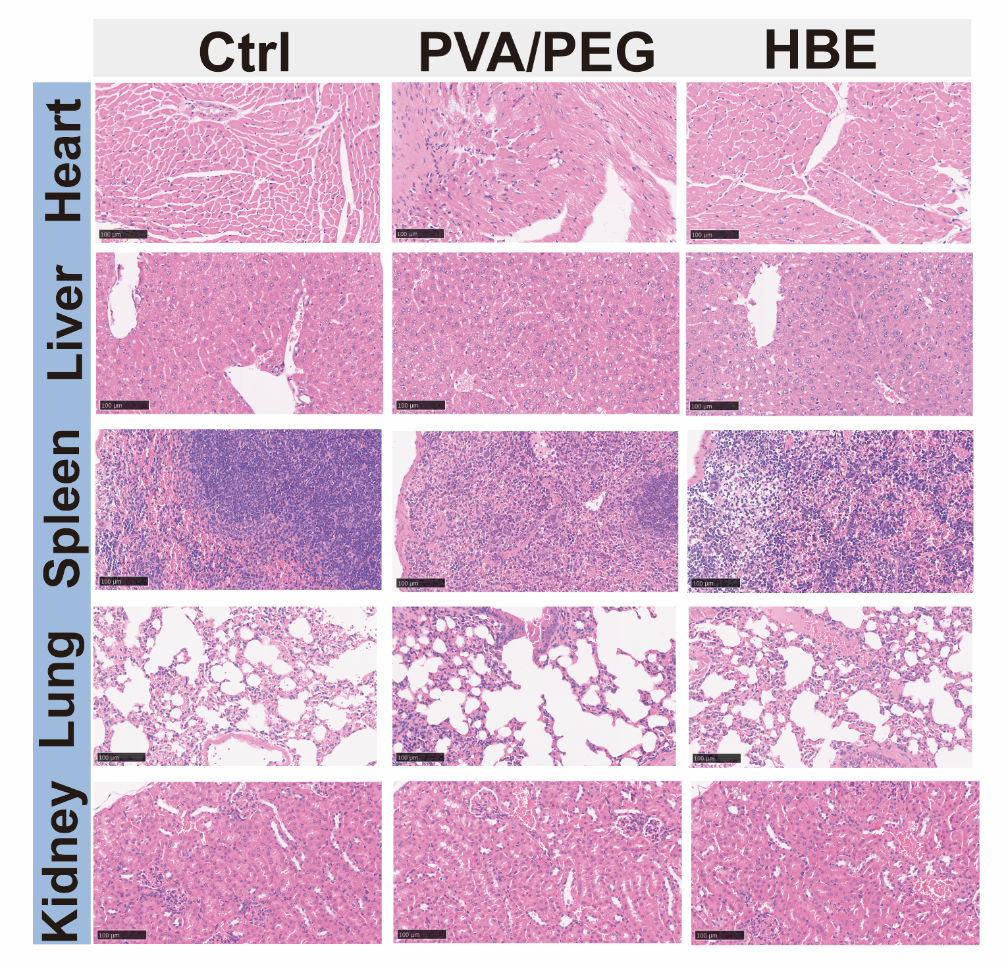
Fig. S5 presents the biocompatibility assessment of major organs after subcutaneous implantation, demonstrating the excellent biocompatibility of the HBE hydrogel electrodes.

Fig. S5 Biocompatibility evaluation of the major organs (heart, liver, spleen, lung, and kidney) stained with hematoxylin and eosin (H&E) from rat after different treatment groups（3 days after subcutaneous implantation). Scale bar is equal to 100 μm.

Fig. S6 shows the representative EEG signals in 295 to 299 seconds with HBE electrodes


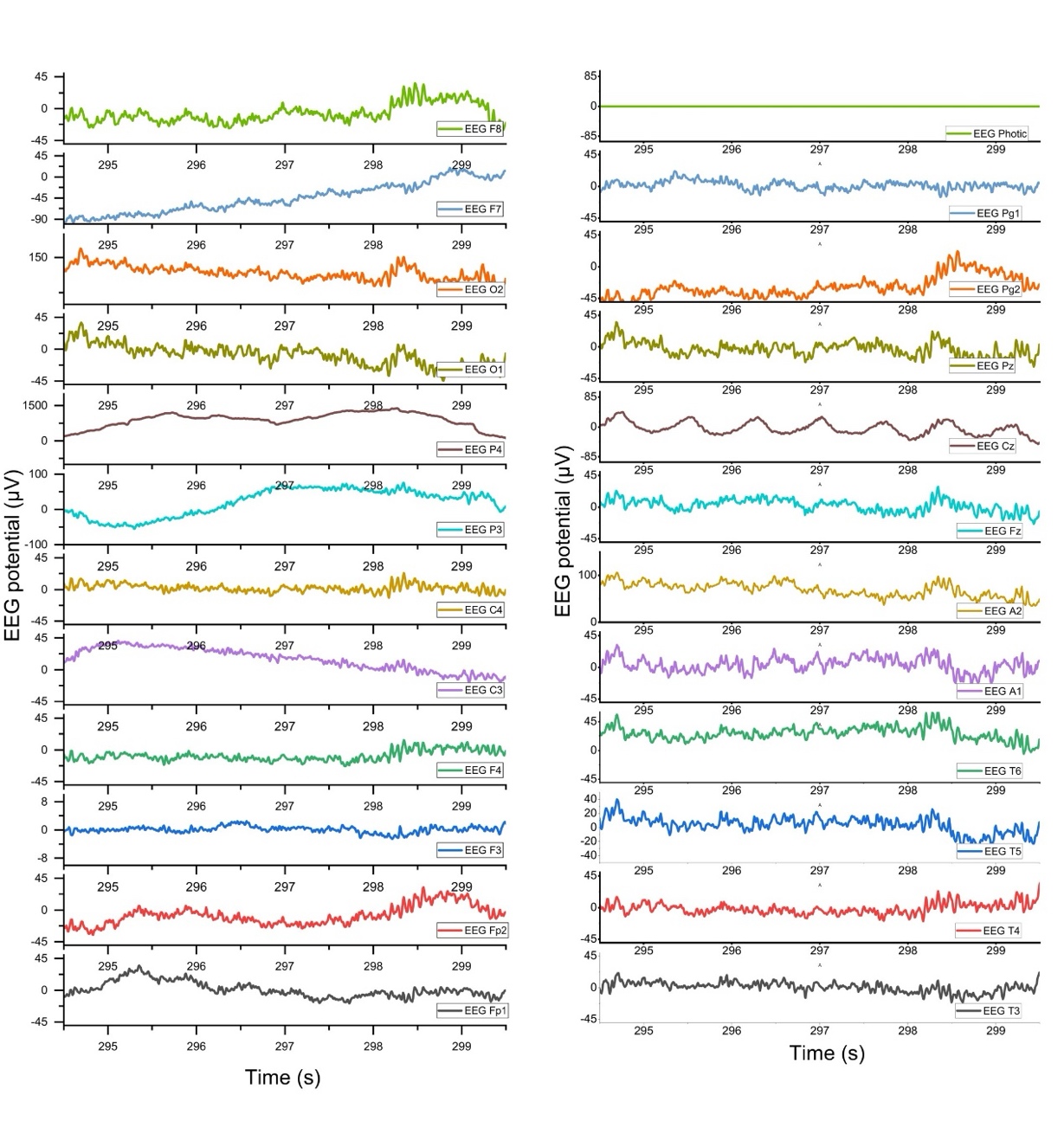


Fig. S6 Representative EEG signals in 295 to 299 seconds with HBE electrodes

Fig. S7 shows the contact impedance between brain electrode and scalp observed during EEG signal detection


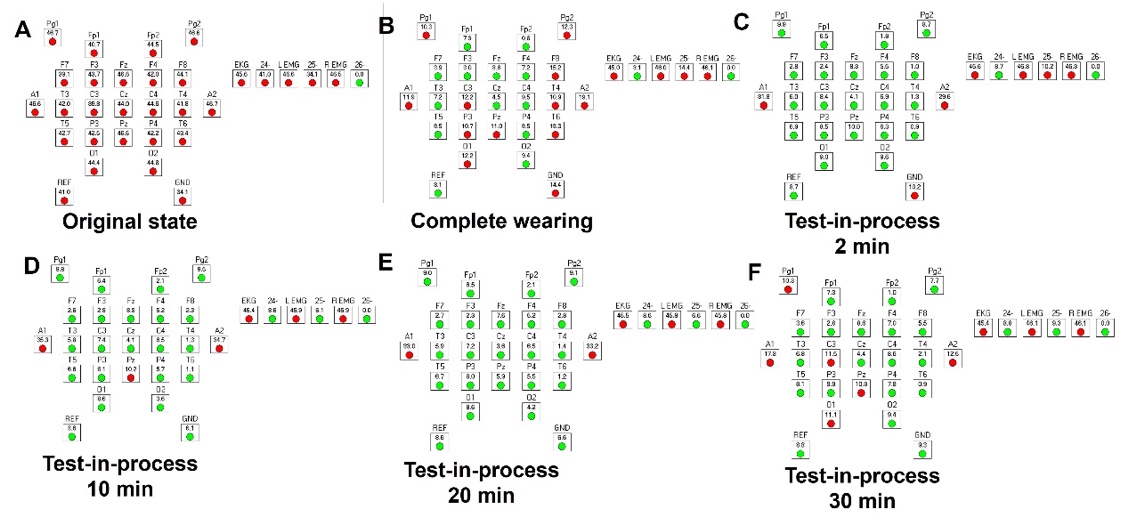


Fig. S7 Changes of impedance with signal acquisition time during EEG collection by HBE
